# The Protection of Crocin Against Ulcerative Colitis and Colorectal Cancer via Suppression of NF-κB-Mediated Inflammation

**DOI:** 10.3389/fphar.2021.639458

**Published:** 2021-03-18

**Authors:** Shanshan Teng, Jie Hao, Hui Bi, Congcong Li, Yongfeng Zhang, Yaqin Zhang, Weiwei Han, Di Wang

**Affiliations:** ^1^ School of Life Sciences, Jilin University, Changchun, China; ^2^ Department of Anesthesiology, Hospital of Stomatology, Jilin University, Changchun, China

**Keywords:** crocin, ulcerative colitis, colorectal cancer, anti-inflammation, antitumor, NF-κB

## Abstract

**Background:** In China, the incidence of ulcerative colitis (UC) is increasing every year, but the etiology of UC remains unclear. UC is known to increase the risk of colorectal cancer (CRC). The aim of this study was to investigate the protective effects of crocin against UC and CRC in mouse models.

**Methods:** Crocin was used to treat the dextran sodium sulfate (DSS)-induced UC mice for 3 weeks, and Apc^MinC^/Gpt mice with colorectal cancer for 8 weeks. Proteomics screening was used to detect changes in the protein profiles of colon tissues of UC mice. Enzyme-linked immunosorbent assays and western blot were used to verify these changes.

**Results:** Crocin strongly reduced the disease activity index scores of UC mice, and improved the pathological symptoms of the colonic epithelium. The anti-inflammatory effects of crocin were indicated by its regulation of the activity of various cytokines, such as interleukins, via the modulation of nuclear factor kappa-B (NF-κB) signaling. Crocin significantly suppressed tumor growth in Apc^MinC^/Gpt mice and ameliorated pathological alterations in the colon and liver, but had no effects on spleen and kidney. Additionally, crocin significantly decreased the concentrations of interleukins and tumor necrosis factor-α in the sera and colon tissues, suggesting its anti-inflammatory effects related to NF-κB signaling. Finally, 12-h incubation of SW480 cells with crocin caused cell cycle arrest, enhanced the apoptotic rate, promoted the dissipation of mitochondrial membrane potential, and the over-accumulation of reactive oxygen species. From the theoretical analyses, phosphorylated residues on S536 may enhance the protein-protein interactions which may influence the conformational changes in the secondary structure of NF-κB.

**Conclusion:** The protective effects of crocin on UC and CRC were due to its suppression of NF-κB-mediated inflammation.

## Introduction

Ulcerative colitis (UC) is a chronic and relapsing inflammatory intestinal disease, and typical UC symptoms are bloody diarrhea, mucus discharge, acute pain and weight loss (Baumgart and Sandborn, 2007; Wu and Chen, 2019). Notably, the etiology of UC remains unclear. UC is known to increase the risk of colorectal cancer (CRC) (Zhao et al., 2018), as pro-inflammatory cytokines in UC can promote tumorigenesis by triggering the formation and metastasis of tumor blood vessels.

In China, the incidence of UC is increasing every year, statistical data show that the prevalence rates of Heilongjiang, Xian, Guangzhou, Zhongshan, Hong Kong and Taiwan are 1.77/100,000, 0.42/100,000, 3.44/100,000, 3.14/100,000, 3.06/100,000 and 4.59/100,000 (Lu and Zhao, 2020). With most UC patients having repeated attacks (Pu et al., 2019). Genetic factors, immunologic elements and environmental factors are considered to contribute to the development of UC (Yamamoto-Furusho et al., 2019). In addition, low concentrations of peroxisome proliferator-activated receptor γ (PPAR-γ) have been noted in the colon cells of UC patients (Scaioli et al., 2017), and PPAR-γ suppresses colitis by negatively regulating nuclear factor kappa-B (NF-κB) (Dubuquoy et al., 2003), this finding suggests that there is an important causal relationship between UC and NF-κB-mediated inflammatory pathways. Another link between these conditions is evidenced by the fact that colonic epithelial cells may become cancerous and eventually lead to CRC, which is a common complication of UC. The activation of NF-κB is responsible for the expression of interleukin (IL)-6 (Qiu et al., 2015), which has been recognized as the key promoter in the tumorigenesis of early colitis-associated cancer (Grivennikov et al., 2009). UC patients have a 2.4-fold increased risk of CRC (Yang et al., 2016), and UC-associated CRC constitutes 1% of all CRC cases (Bopanna et al., 2017).

At present, surgery, radiotherapy and chemotherapy are the common therapeutic strategies for CRC (Lin et al., 2018). In addition, aminosalicylic acid preparations, glucocorticoids and immunosuppressive agents are the three major types of drugs used clinically in the treatment of UC; however, these drugs have side effects, some of which are serious, which greatly limits their application (Wu and Chen, 2019). Similarly, 5-fluorouracil is commonly used for treating CRC, but resistance to this drug can occur during chemotherapy (Wang et al., 2017b). Therefore, the aim of this study was to explore an agent that may have the potential to treat UC and CRC and to prevent the development of CRC from UC.

Among the therapeutic agents approved by the Food and Drug Administration (FDA), 40% are natural ingredients or their derivatives (Al-Hrout et al., 2018). Due to the low toxicity and high efficacy of natural products, natural products have been extensively studied and used to treat various diseases (Amin et al., 2012; Hamza et al., 2016; Ala’a et al., 2017; Al-Dabbagh et al., 2018; Hamza et al., 2018; Al-Dabbagh et al., 2019; El-Dakhly et al., 2020).

Crocin (C_44_H_64_O_24_), the main biologically active compound of saffron, has been reported to show various pharmacological activities (Ashktorab et al., 2019), including anti-inflammatory and anti-cancer activities (Khorasany and Hosseinzadeh, 2016). Saffron threads are the dried stigmas of Crocus sativus L (Iridaceae) and constitute one of the most expensive and valuable spices in the world, leading to it being dubbed “red gold”. Saffron is cultivated in Iran; Mediterranean regions such as Italy, Spain and Greece; Northern Africa India (Kashmir) and some other countries in Europe and Asia (Yilmaz et al., 2010). Notably, saffron was used by the ancient Egyptians to treat gastrointestinal diseases (Khorasany and Hosseinzadeh, 2016).

In modern-day studies, administration of crocin has shown to inhibit dextran sodium sulfate (DSS)-induced UC and suppress the expression of various cytokines such as tumor necrosis factor-α (TNF-α) and NF-κB (Kawabata et al., 2012). Crocin also has anti-inflammatory effects in DSS-induced colitis mice, such that it can be used to prevent or treat colitis (Rezaei et al., 2019), and this activity was shown to be related to its regulation of NF-κB (Khorasany and Hosseinzadeh, 2016). Moreover, the anti-CRC effects of crocin may be related to its anti-proliferative and pro-apoptotic activities (Khorasany and Hosseinzadeh, 2016; Amerizadeh et al., 2018). However, the underlying mechanisms of the protective effects of crocin against UC and CRC have not been systematically investigated.

In this study, the anti-UC and anti-CRC effects of crocin were successfully confirmed by experiments in a human colon adenocarcinoma cell line, SW480, and in DSS-induced UC mice and Apc^MinC^/Gpt mice, where the latter were obtained by point-mutating the adenomatous polyposis coil (APC) gene. Our experiments confirmed that the anti-inflammatory effects of crocin are related to its regulation of NF-κB signaling.

## Materials and Methods

### Cell Culture

SW480 cells, a human colon adenocarcinoma cell line (catalog number: SCSP-5033; Chinese Academy of Sciences, Shanghai, China), were cultured in Dulbecco’s modified Eagle’s medium (DMEM) containing 10% fetal bovine serum (FBS), 1% 100 μg/ml streptomycin and 100 units/ml penicillin (Thermo Fisher Scientific, Inc., Waltham, MA, United States) at 37 C in a CO_2_ incubator.

### Cell Viability Assay

SW480 cells were seeded into 96-well plates at a density of 8 × 10^3^ cells/well. After incubation at 37 C overnight, cells were treated with 0.4, 0.8, 2, 3 and 6 mM of crocin (catalog number: B21336; Shanghai Yuanye Biological Technology Co., Ltd., Shanghai, China) for 24 h at 37 C. Thereafter, 5 μl of 3-(4, 5-dimethyl-2-thiazolyl)-2,5-diphenyl-2-*H*-tetrazolium bromide (MTT) (Shanghai Yuanye Biological Technology Co., Ltd., Shanghai, China) was added to each well, to obtain a final concentration of 5 mg/ml MTT. The plates were then incubated at 37 C for a further 4 h, at which point the supernatant was aspirated, 100 μl of dimethyl sulfoxide (catalog number: DH105-2; Beijing Dingguo Changsheng Biotechnology Co., Ltd., Beijing, China) was added to each well, and finally the absorbance of the plates was measured at 490 nm using a Synergy™ 4 Microplate Reader (Biotek, Winooski, Vermont, United States).

### Cell Cycle, Cell Apoptosis and Mitochondrial Membrane Potential (MMP) Detection

SW480 cells were seeded into six-well plates at a density of 2 × 10^5^ cells/well, and the plates were incubated at 37 C overnight. Thereafter, cells were treated with 2.5 mM or 5 mM crocin and incubated at 37 C for a further 12 h.

For cell cycle detection, collected cells were washed with pre-cooled phosphate buffered saline (PBS) and then incubated with 70% pre-cooled ethanol for more than 3 h. The cells were then exposed to Muse™ Cell Cycle Reagent (catalog number: MCH100106; EMD Millipore Corp., Billerica MA, United States) at room temperature for 30 min in darkness. A Muse™ Cell Analyzer (EMD Millipore Corp., Billerica MA, United States) was then used to detect the cell cycle conditions of the cells.

For cell apoptosis detection, collected cells were resuspended in DMEM containing 1% FBS and 1% bovine serum albumin (BSA) at a final concentration of 1 × 10^6^ cells/ml. Cells were incubated with the Muse™ Annexin V and Dead Cell Reagent (catalog number: MCH100105; EMD Millipore Corp., Billerica MA, United States) at room temperature for 20 min in darkness. A Muse™ cell analyzer was then used to detect the apoptotic status of the cells.

For MMP detection, collected cells were treated with mitopotential working fluid at a final concentration of 10 μM (catalog number: MAK160; Sigma-Aldrich, St. Louis, Missouri, United States) and then incubated at 37 C in darkness for 20 min. Thereafter, cells were washed three times with PBS, and then the MMP changes of SW480 cells were analyzed using a Muse™ cell analyzer.

### Animal Experimental Protocol

The experiment was approved by the Institutional Animal Ethics Committee of Jilin University (SY201905009 and SY201905004). C57BL/6 J mice (male, 7–8 weeks old, 22–25 g) (catalog number: cs-005) purchased from Liaoning Changsheng Biotechnology Co., Ltd (Liaoning, China) and Apc^MinC^/Gpt mice (male, 4–8 weeks old, 20–25 g) (catalog number: T001457) purchased from GemPharmatech Co., Ltd. (Jiangsu, China) were kept in a temperature- and humidity-controlled room at 22 ± 1 C and 50–65% humidity, with no convective wind and guaranteed sunshine for 12 h each day. Mice were provided with clean water and food each day, and their litter was cleaned daily.

Sixty C57BL/6 J mice were adaptively fed for 1 week, and then randomly divided into five groups (*n* = 12 per group). All 48 treatment-group mice were allowed ad libitum access to 3.0% solution of DSS in water (catalog number: S14048; Shanghai Yuanye Biological Technology Co., Ltd., Shanghai, China) for 1 week. Subsequently, model mice (*n* = 12) were orally treated with normal saline, positive control mice (*n* = 12) were orally treated with 0.6 g/kg of sulfasalazine (SASP) (catalog number: BP779; Sigma-Aldrich, St. Louis, Missouri, United States), and crocin-treated mice were orally treated with 10 mg/kg (*n* = 12) and 30 mg/kg (*n* = 12) crocin once per day for 3 weeks. All mice continued to have ad libitum access to 3.0% DSS every second day for a further 3 weeks. The control mice (*n* = 12) (entitled CTRL) were orally treated with normal saline once per day for 3 weeks.

Twenty Apc^MinC^/Gpt mice were properly fed for 9 weeks, and then randomly divided into two groups (*n* = 10 per group). One of these was designated the control group, and these mice were treated with normal saline (0 mg/kg of crocin); the other was designed the treatment group, and these mice were treated orally with 30 mg/kg of crocin once per day for 8 weeks.

At the conclusion of the experiment, all mice were euthanized by sodium barbiturate injection. Blood samples were collected from mouse caudal veins, and organs (colon, liver, kidney, and spleen) were collected for subsequent biochemical and pathological analysis.

### Disease Activity Index (DAI) Score of Mice With UC

The body weight, stool consistency and bleeding of mice with UC were recorded daily, and their DAI scores were measured according to the Hamamoto standard (Hamamoto et al., 1999).

### Proteomics

A protein sample solution was obtained from the colon of mice with UC, quantified by using a bicinchoninic acid (BCA) kit, and then hydrolyzed with lyase. The resulting peptide samples were detached and then analyzed by liquid chromatography-tandem mass spectrometry, and the results were processed using MaxQuant (1.5.6.0). The UNIPROT database (Uniprot_mouse_1206_09) was used to obtain protein sequences, and the protein sequences and their respective reverse bait sequences were used in the MaxQuant search. Corresponding differentially expressed proteins were identified from statistical analysis of standardized quantitative results. Proteins with different expression folds (ratio A/B > 1.5 or ratio A/B < 0.66) were defined as significantly different. Finally, Gene Ontology (GO) and Kyoto Encyclopedia of Genes and Genomes (KEGG) pathway analyses were performed to determine protein interactions.

### Enzyme-Linked Immunosorbent Assay (ELISA)

The colon tissues of mice with UC and CRC were homogenized with physiological saline, and the protein concentration of homogenates was measured using a Pierce™ BCA Protein Assay Kit (catalog number: 23225; Thermo Scientific™, Shanghai, China). The concentrations of IL-1β (catalog number: KT2040-A), IL-2 (catalog number: KT2795-A), IL-4 (catalog number: KT2165-A), IL-6 (catalog number: KT2163-A), IL-15 (catalog number: FY2172-A), IL-17 (catalog number: KT2170-A), IL-18 (catalog number: KT2169-A), inducible nitric oxide synthase (iNOS) (catalog number: KT2454-A), cyclooxygenase-2 (COX-2) (catalog number: KT2356-A), TNF-α (catalog number: KT2132-A) and interferon-α (IFN-α) (catalog number: FY2366-A; Jiangsu Kete Biotechnology Co., Ltd., Yancheng, Jiangsu, China) in the colon and serum samples from mice with UC and CRC were determined according to the manufacturer’s instructions.

### Hematoxylin and Eosin (H&E) Staining

Fresh samples of colon, liver, kidney, and spleen tissues from mice with UC and CRC were fixed in 10% formalin, and the fixed tissues were then dehydrated by successive exposure to 30, 50, 70, 80, 95 and 100% ethanol. The dehydrated tissues were then immersed in xylene, embedded in wax, and cut into 5 μm sections. The sections were dewaxed using xylene, and then rehydrated by successive treatment with 100, 95, 80, 70, 50 and 30% ethanol. Finally, the rehydrated sections were stained by H&E and observed under a medical biological microscope (catalog number: BX51; Olympus, Japan).

### Western Blot

Proteins were extracted from colon tissues using radio immunoprecipitation assay (catalog number: R0010; Beijing Solarbio Science and Technology Co., Ltd., Beijing, China) buffer containing 1% protease inhibitor cocktail (catalog number: P8340; Sigma-Aldrich, St. Louis, MO, United States) and 2% phenylmethanesulfonyl fluoride (catalog number: P7626; Sigma-Aldrich, St. Louis, MO, United States). Protein concentrations were measured using the Pierce™ BCA Protein Assay Kit. Protein samples (40 μg/μl) were separated using 12% sodium dodecyl sulfate polyacrylamide gel electrophoresis, and transferred to a 0.45 μm polyvinylidene difluoride membrane (Merck Millipore, Billerica, MA, United States). The loaded membranes were incubated with 5% BSA solution at 4 C for 5 h, and then incubated at 4 C overnight with the following primary antibodies: total- (T-) inhibitor of nuclear factor kappa-B kinase subunit (α/β) [IKK (α/β)] (75/87 kDa) (dilution: 1:2000) (catalog number: bs-10123 R), B-cell lymphoma-2 (Bcl-2) (26 kDa) (dilution: 1:2000) (catalog number: bsm-33047M; Beijing Bioss Biotechnology Co., Ltd., Beijing, China), and phospho- (P-) inhibitor of nuclear factor kappa-B-α (IκBα) (40 kDa) (dilution: 1:2000) (catalog number: bs-18129R), P-IKK (α/β) (85/87 kDa) (dilution: 1:1000) (catalog number: ab194528; Abcam, Cambridge, MA, USA), Bcl-2 associated X protein (Bax) (21 kDa) (dilution: 1:2000) (catalog number: ab32503), T-IκBα (35 kDa) (dilution: 1:2000) (catalog number: ab32518), T-NF-κB p65 (65 kDa) (dilution: 1:2000) (catalog number: ab16502), P-NF-κB p65 (65 kDa) (dilution: 1:4000) (catalog number: ab86299), UBX domain-containing protein 1 (UBXN1) (35 kDa) (dilution: 1:2000) (catalog number: ab154265), Dedicator of cytokinesis 1 (DOCK1) (215 kDa) (dilution: 1:2000) (catalog number: ab76927), and glyceraldehyde-3-phosphate dehydrogenase (GAPDH) (37 kDa) (dilution: 1:2000) (catalog number: E-AB-20032; Elabscience Biotechnology Co., Ltd., Wuhan, China).

Subsequently, the membranes were washed and then incubated with goat anti-rabbit IgG (H + L) [peroxidase/horseradish peroxidase (HRP) conjugated] (catalog number: E-AB-1003; Elabscience Biotechnology Co., Ltd., Wuhan, China) or goat anti-mouse IgG (H + L) (peroxidase/HRP conjugated) (catalog number: E-AB-1001) at 4 C for 3–4 h. The membranes were then washed again, and treated with NcmECL Ultra (catalog number: P10200; New Cell and Molecular Biotech Co., Ltd., Suzhou, China), a high–sensitivity electro-chemi-luminescence (ECL) reagent. The protein band was then visualized on an imaging system (catalog number: Tanon 5200; Shanghai Tianneng Technology Co., Ltd., Shanghai, China). The imaging results were analyzed using Image–J software (National Institutes of Health, Bethesda, MD, United States).

### Statistical Analysis

All values are expressed as the means ± standard deviation (SD). A one-way analysis of variance (ANOVA) was used to detect statistical significance, and post-hoc multiple comparisons (Tukey’s test) were performed using SPSS 16.0 software (IBM corporation, Armonk, NY, United States). A *p* value of <0.05 was considered to be statistically significant.

### Theoretical Study

The 3D structure of crocin was optimized by Gaussian 09 software (Ali et al., 2020) at 6-31G* set (Ye et al., 2020). And then crocin was docked to NF-κB (the 3D structure of NF-κB was built by Swiss model software^1^) using AutoDock 4.2 software (Liu et al., 2019; Umesh et al., 2020). The current version of AutoDock, using the lamarckian genetic algorithm and empirical free energy scoring function, typically will provide reproducible docking results for ligands with approximately 10 flexible bonds. Four systems (P-NF-κB, T-NF-κB, P-NF-κB + crocin, and T- NF-κB + crocin) were performed molecular dynamics simulations using Amber 16 software (Wang et al., 2017a; Lee et al., 2017b). Protein network centrality analysis, which can compute weighted centrality measures among ligand binding to protein, were performed with the cytoscape plugin RINalyzer (Doncheva et al., 2011; Zhu et al., 2018).

## Results

### The Anti-UC Effects of Crocin

**FIGURE 1:**
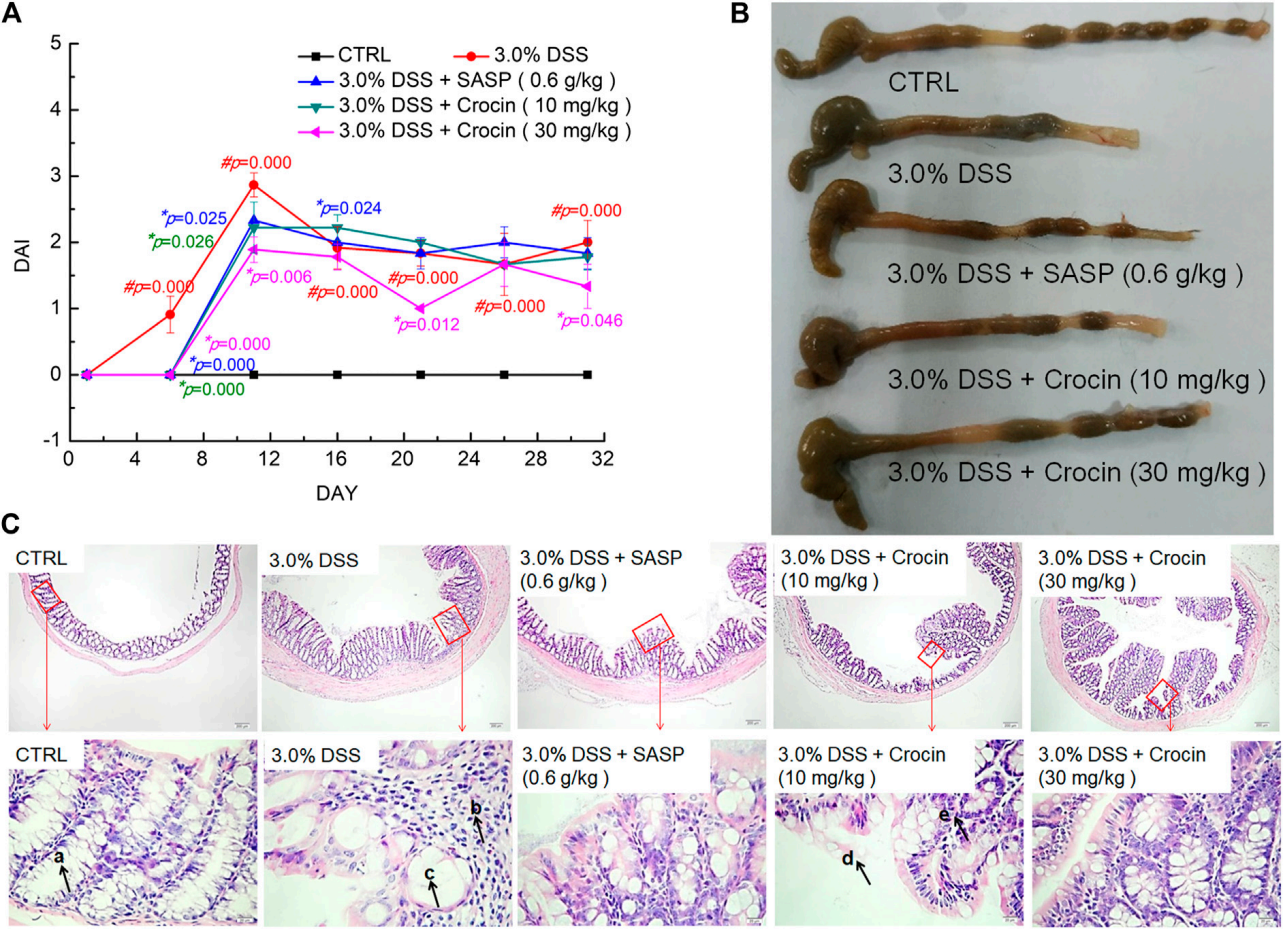
The protection of crocin against UC caused by DSS. **(A)** Crocin reduced the DAI scores increased by DSS. **(B)** Crocin improved the pathological symptoms of colonic epithelium in UC mice. **(C)** Histopathological observation of colon tissues of UC mice (40×, scale bar: 200 μm) (400×, scale bar: 20 μm) (a. intestinal crypts, b. large numbers of scattered lymphocytes, c. crypt goblet cells are damaged and reduced, d. damage to the epithelium, e. few lymphocytes.) Data are presented as the mean ± SD (*n* = 12) and analyzed via a one-way ANOVA test followed by post-hoc Tukey's multiple comparison tests. The significant changes compared with CTRL mice marked as *#p* value, and the significant changes compared with 3.0% DSS only treated mice marked as *p value.

A DAI score can be used to assess the severity of UC (Wu and Chen, 2019). We found that the ad libitum access to DSS-dosed water significantly increased the DAI score (*p* = 0.000) of mice, and that this was reversed after 32 days treatment with crocin or SASP (*p* < 0.05) (Figure 1A).

The colonic epithelial tissues of CTRL mice were smooth and ductile; in contrast, ulcers and edema were apparent in the colonic epithelial tissues of vehicle-treated UC mice, and these were markedly improved after treatment with crocin or SASP (Figure 1B). The colons of crocin- or SASP-treated mice were substantially longer than those of model mice (Figure 1B). Notably, there were no significant changes in the spleen, liver and kidney structures in any experimental mice (Supplementary Figure S1). There were numerous pathological alterations in the colon tissues of all UC mice, such as irregularly arranged and damaged surface epithelia, branchless, distorted and loosely arranged goblet cells in the deep part of the crypt, and a large number of scattered lymphocytes in the lamina propria with concurrent epithelial loss, compared with the colons of CTRL mice that were normal in appearance. However, these pathologic alterations were greatly attenuated in the crocin- and SASP-treated UC mice (Figure 1C).

### The Anti-Inflammatory Effects of Crocin in Mice with UC Occur Via Regulation of NF-κB Signaling

**FIGURE 2:**
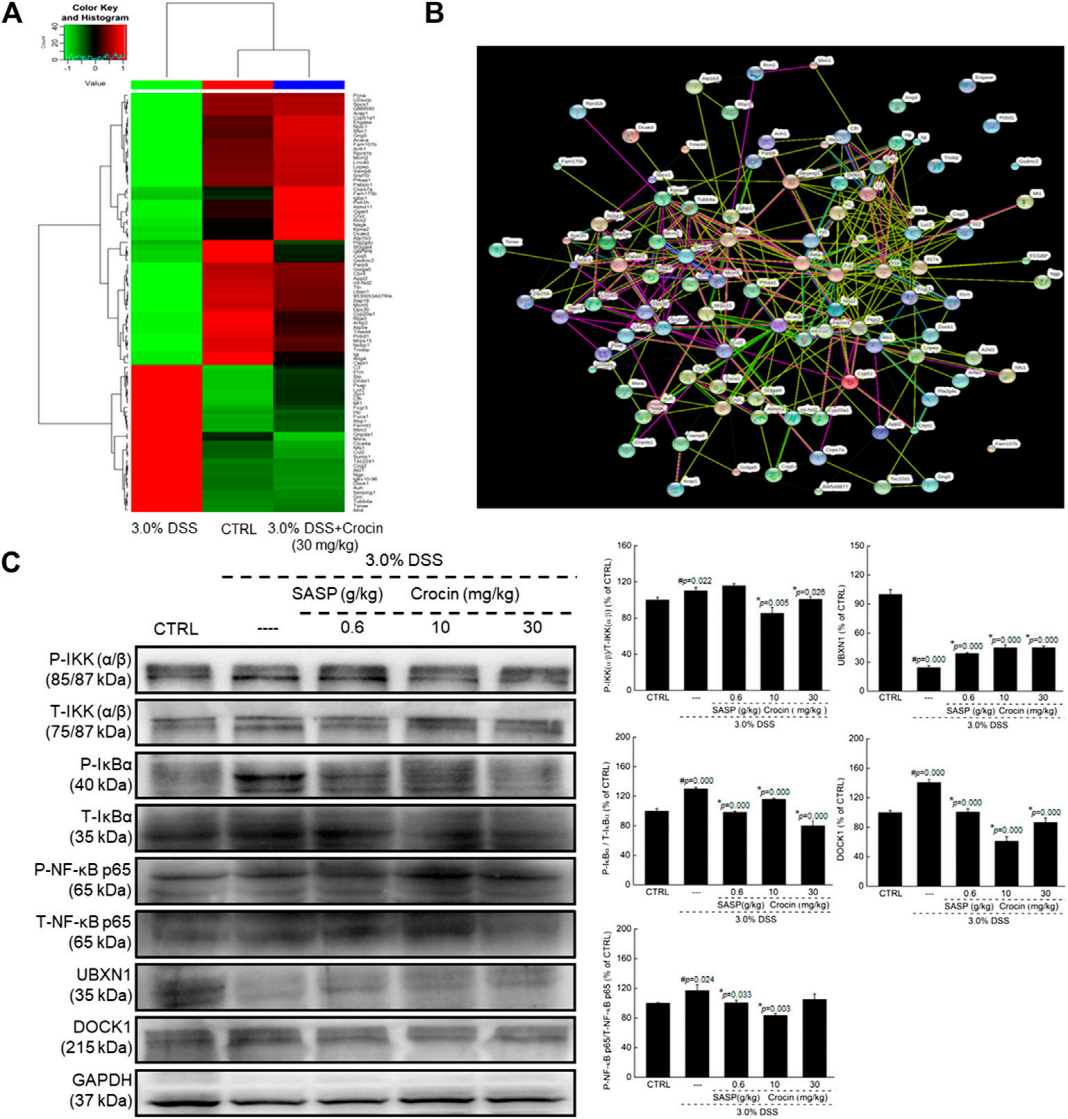
Crocin broadly regulated protein expression in colon tissues analyzing by proteomics in mice with UC, and showed anti-inflammation in mice with UC via regulating NF-κB signaling pathway. **(A)** Heat map of obvious change factors (*n* = 3). **(B)** The relationship between proteins processed through STRINGdb (*n* = 3). **(C)** Compared with 3.0% DSS only treated mice, crocin suppressed the phosphorylation of IKK(α/β), IκBα and NF-κB, and the expression levels of DOCK1, and enhanced the expression levels of UBXN1 in colon lysis. Data are presented as the mean ± SD (*n* = 3) and analyzed via a one-way ANOVA test followed by post-hoc Tukey's multiple comparison tests. The significant changes compared with CTRL mice marked as *#p* value, and the significant changes compared with 3.0% DSS only treated mice marked as *p value.

Ninety-four factors related to NF-κB signaling during the development of UC were screened by protein profiling of the colon tissues of experimental mice (Figure 2A), of which 68 protein factors finally met the trend (Supplementary Table S1). Additionally, based on the analysis of protein interactions, 101 types of proteins related to NF-κB signaling were identified (Figure 2B). The changes in the concentrations of factors such as iNOS, TNF-α, COX-2 and interleukins (ILs) among experimental groups were further confirmed by ELISA. In the colon tissues of UC mice, extremely high concentrations of IL-1β (*p* = 0.000), IL-2 (*p* = 0.006), IL-6 (*p* = 0.000), IL-17 (*p* = 0.002), iNOS (*p* = 0.006) and COX-2 (*p* = 0.007) were observed, and the concentrations of all of these factors were substantially reduced by 21-days crocin treatment (*p* < 0.01) (Table 1). Similarly, SASP significantly regulated the colonic concentrations of detected cytokines (*p* < 0.05), except for those of TNF-α (Table 1). Crocin treatment also markedly reduced the concentrations of inflammatory species factors in the sera of UC mice, notably IL-1β (*p* < 0.01), IL-2 (*p* = 0.000), IL-6 (*p* = 0.000), iNOS (*p* < 0.01), TNF-α (*p* < 0.01) and COX-2 (*p* = 0.018). In addition, SASP reduced the serum concentrations of IL-1β (*p* = 0.000), IL-2 (*p* = 0.000), IL-6 (*p* = 0.000), iNOS (*p* = 0.003) and TNF-α (*p* = 0.000), but not those of IL-17 and COX-2, in UC mice (Table 1).

**TABLE 1:** The effect of crocin on levels of cytokines related to inflammation in serum and colon of mice with UC.

		CTRL	DSS			
			−−	SASP (g/kg)	Crocin (mg/kg)	
				0.6	10	30
Serum	iNOS (μmol/L)	16.90 ± 2.79	22.52 ± 4.38^*#p*=0.027^	14.30 ± 1.88^*p=0.003^	13.96 ± 1.34^*p=0.004^	13.74 ± 1.44^*p^ ^=0.009^
	IL−1β (ng/L)	27.18 ± 5.48	53.99 ± 10.62^*#p*=0.000^	5.49 ± 2.07^*p=0.000^	30.48 ± 6.24^*p=0.001^	10.79 ± 1.51^*p=0.000^
	IL−2 (ng/L)	1.50 ± 0.59	2.59 ± 0.50^*#p*=0.006^	1.11 ± 0.29^*p=0.000^	1.37 ± 0.42 ^*p=0.000^	1.41 ± 0.27^*p^ ^=0.000^
	IL−6 (pg/ml)	32.08 ± 6.05	64.91 ± 9.58^*#p*=0.001^	28.59 ± 7.07^*p=0.000^	16.20 ± 1.35^*p=0.000^	8.08 ± 1.00^*p^ ^=0.000^
	IL−17 (pg/ml)	163.59 ± 27.29	169.84 ± 30.55	149.64 ± 28.11	155.00 ± 25.83	153.21 ± 30.40
	TNF−α (ng/L)	0.33 ± 0.05	0.57 ± 0.11^*#p*=0.002^	0.22 ± 0.05^*p=0.000^	0.26 ± 0.05^*p=0.001^	0.11 ± 0.02^*p^ ^=0.000^
	COX−2 (ng/L)	50.48 ± 9.50	59.72 ± 8.77	58.34 ± 7.64	56.44 ± 9.50	49.16 ± 8.25^*p^ ^=0.018^
Colon	iNOS (μmol/g)	6.00 ± 1.07	9.39 ± 1.78 ^*#p*=0.006^	5.51 ± 1.05^*p=0.007^	5.07 ± 0.71^*p=0.000^	2.20 ± 0.39^*p=0.000^
	IL−1β (ng/g)	33.91 ± 5.08	64.26 ± 11.32 ^*#p*=0.000^	21.79 ± 2.82^*p=0.000^	31.71 ± 4.82^*p=0.000^	14.49 ± 1.69^*p=0.000^
	IL−2 (ng/g)	0.97 ± 0.21	1.59 ± 0.44 ^*#p*=0.006^	0.66 ± 0.06^*p=0.003^	0.82 ± 0.16^*p=0.004^	0.40 ± 0.06^*p=0.000^
	IL−6 (pg/mg)	38.46 ± 6.87	73.02 ± 13.51 ^*#p*=0.000^	27.64 ± 4.31^*p=0.000^	32.74 ± 5.84^*p=0.000^	13.01 ± 2.49^*p=0.000^
	IL−17 (pg/mg)	68.92 ± 11.61	103.99 ± 16.85 ^*#p*=0.002^	47.16 ± 8.33^*p=0.000^	56.17 ± 8.66^*p=0.000^	29.23 ± 5.08^*p=0.000^
	TNF−α (ng/g)	1.12 ± 0.05	1.18 ± 0.15	1.16 ± 0.16	0.98 ± 0.18^*p=0.032^	0.96 ± 0.16^*p=0.023^
	COX−2 (ng/g)	18.23 ± 3.37	25.31 ± 3.91 ^*#p*=0.007^	15.70 ± 5.53^*p=0.018^	14.71 ± 3.67^*p=0.004^	7.03 ± 0.79^*p=0.000^

Data are presented as the mean ± SD (*n* = 12) and analyzed via a one−way ANOVA test followed by post−hoc Tukey's multiple comparison tests. The significant changes compared with CTRL mice marked as *#p* value, and the significant changes compared with 3.0% DSS only treated mice marked as *p value.

Increased expression of P-IKK (α/β) (*p* = 0.022), P-IκBα (*p* = 0.000), P-NF-κB (*p* = 0.024) and DOCK1 (*p* = 0.000) were noted in the colonic tissues of UC mice, as were reduced expression of UBXN1 (*p* = 0.000). Both SASP and crocin reversed these expression-level alterations (*p* < 0.05) (Figure 2C).

### The Anti-CRC Effects of Crocin in Mice With CRC

**FIGURE 3:**
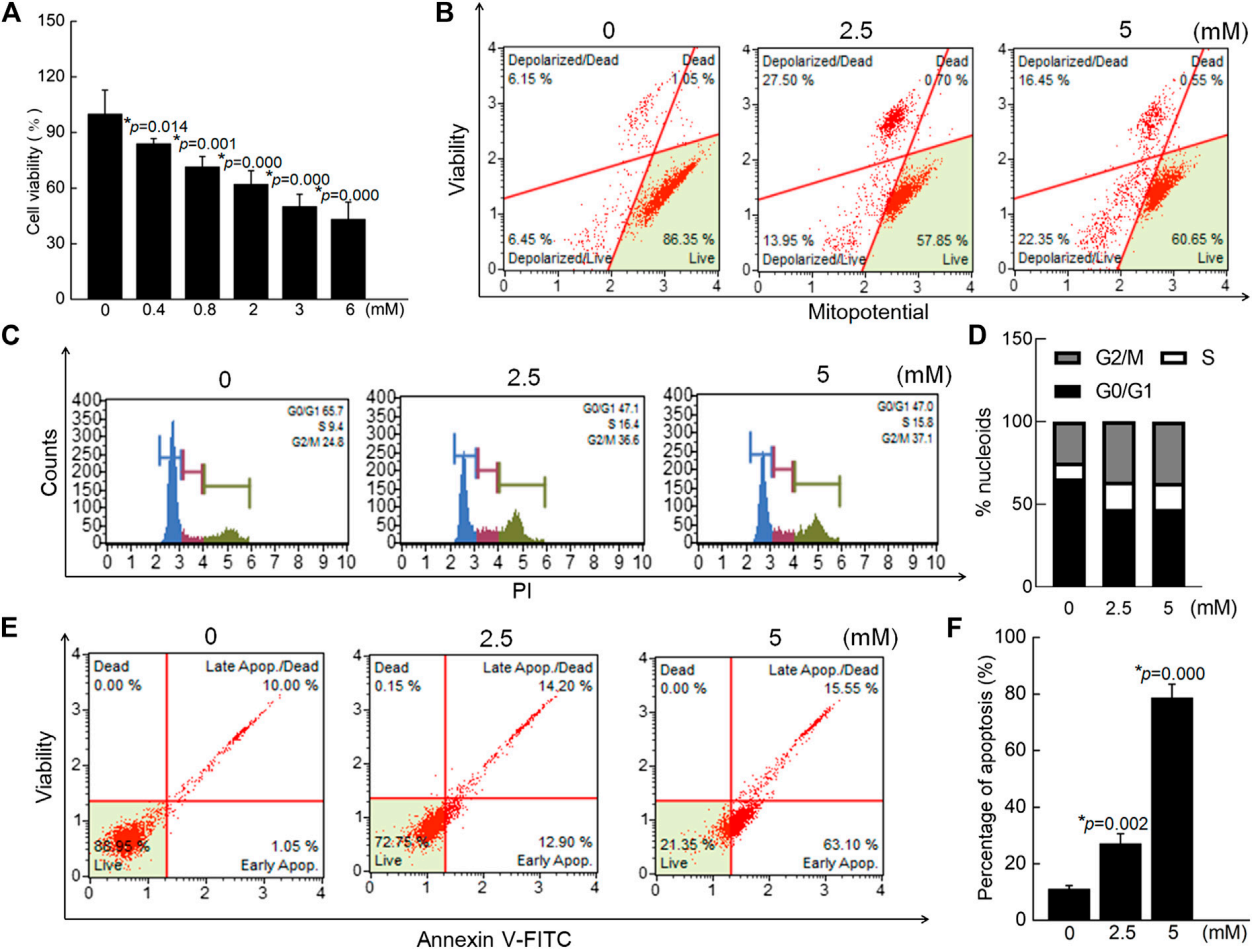
Crocin caused the apoptosis in SW480 cells via modulating the mitochondrial function. **(A)** Crocin suppressed the cell viability of SW480 cells. **(B)** Crocin promoted the dissipation of MMP in SW480 cells. **(C)** Crocin caused the cell cycle arrest. **(D)** is a quantitative analysis of cell cycle. **(E)** Crocin caused the apoptosis in SW480 cells. **(F)** is a quantitative analysis of apoptosis in SW480 cells. Data are presented as the mean ± SD (*n* = 6), and analyzed via a one-way ANOVA test followed by post-hoc Tukey's multiple comparison tests. The significant changes compared with 0 mM of crocin treated cells marked as *p value.

In SW480 cells, 24-h exposure to crocin caused significant reductions in cell viability, corresponding to a 24 h IC_50_ of 3.68 mM (*p* < 0.05) (Figure 3A). Crocin caused 41.45% (2.5 mM) and 38.80% (5 mM) dissipation of MMP in SW480 cells (*vs.* 12.60% in 0 mM crocin) (Figure 3B). Incubation of cells with crocin for 12 h caused cell cycle arrest (Figures 3C,D) and resulted in 27.10% (2.5 mM) (*p* = 0.002) and 78.65% (5 mM) (*p* = 0.000) apoptosis in SW480 cells (*vs.* 11.05% in 0 mM crocin) (Figures 3E,F).

APC mutations occur in the DNA of most CRC patients and can be detected in most sporadic colorectal tumors. Mice with APC mutations can also spontaneously form intestinal tumors (Asadi et al., 2017), and Apc^MinC^/Gpt mice are therefore useful for studying the formation of intestinal tumors. Compared with vehicle-treated mice with CRC, crocin-treated mice displayed markedly increased body weights on day 56 (*p* = 0.044), and increased spleen index (*p* = 0.002), and reduced indexes of liver (*p* = 0.009), and kidney (*p* = 0.044) (Supplementary Table S2).

**FIGURE 4:**
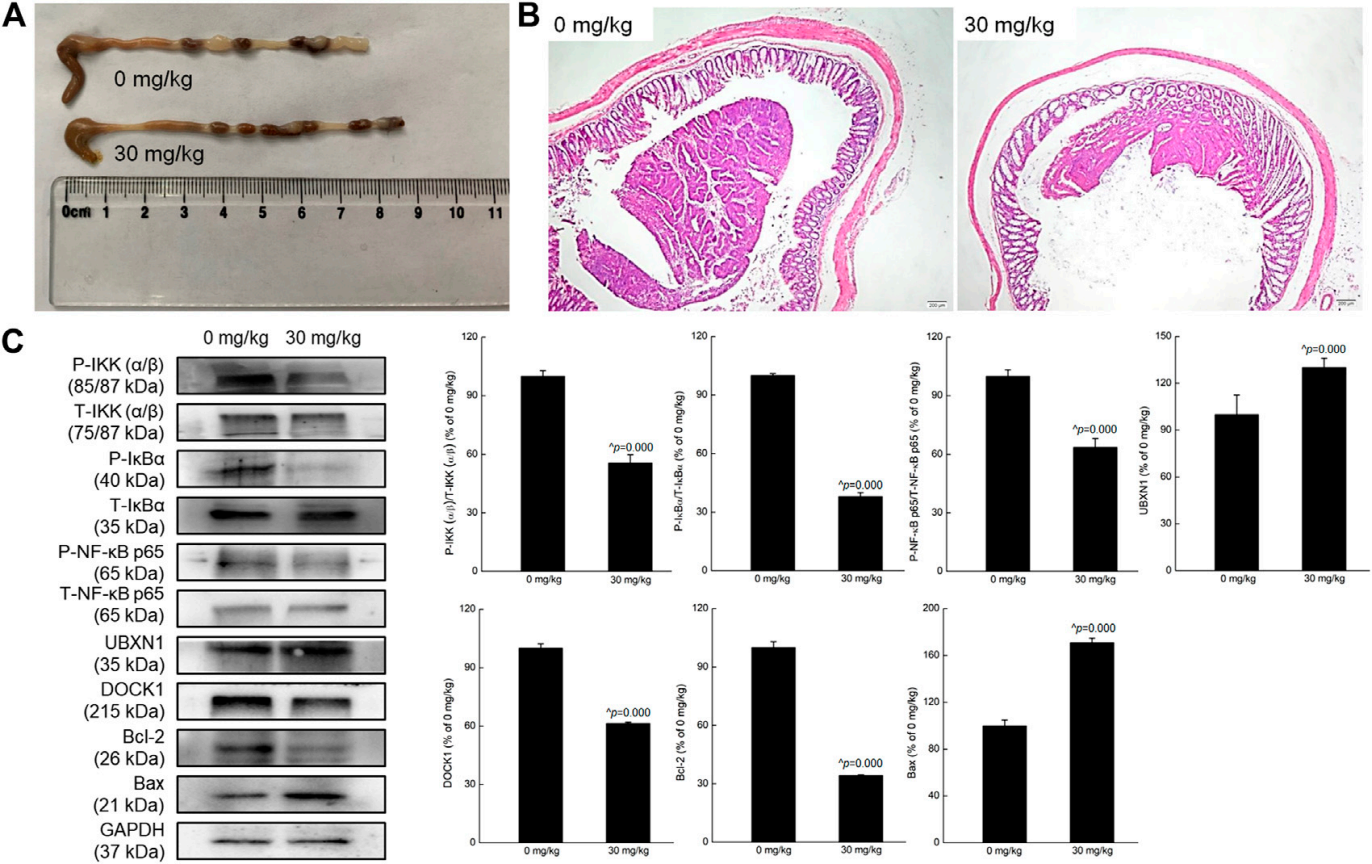
Crocin suppressed the tumor growth in mice with CRC, showed anti-inflammation in mice with CRC via regulating NF-κB signaling pathway. **(A)** Crocin improved the pathology of colon in CRC mice, suggesting by the reduced tumor number and tumor size. **(B)** Histopathological observation of colon tissues of CRC mice with treatment of crocin (40×, scale bar: 200 μm) (n = 3). **(C)** Compared with vehicle-treated mice, crocin suppressed the phosphorylation of IKK (α/β), IκBα and NF-κB, and the expression levels of DOCK1 and Bcl-2, and enhanced the expression levels of UBXN1 and Bax in colon lysis. Data are presented as the mean ± SD (*n* = 3) and analyzed via a one-way ANOVA test followed by post-hoc Tukey's multiple comparison tests. The significant changes compared with vehicle-treated (0 mg/kg of crocin) mice marked as *^p* value.

Far fewer colonic tumors and longer intestines were noted in crocin-treated mice compared with vehicle-treated mice (Figure 4A). Pathological examination also showed that crocin greatly reduced the volume of intestinal cavity-occupying tumors in the colon tissues of mice (Figure 4B). There were no significant differences in the organ structures of spleen and kidney between vehicle-treated- and crocin-treated- mice (Supplementary Figure S2). Compared with vehicle treatment in CRC, crocin treatment reversed liver damage, such as the degeneration of hepatocyte vacuoles round the central vein of the hepatic lobules (Supplementary Figure S2).

### The Anti-Inflammatory Effects of Crocin Played Important Roles During its Anti-CRC Activity in Mice

In CRC mice, 8-weeks crocin administration substantially reduced the concentrations of IL-1β (*p* < 0.01), IL-6 (*p* < 0.01), IL-17 (*p* < 0.05) and TNF-α (*p* < 0.05) and increased those of IL-15 (*p* = 0.000) and IFN-α (*p* = 0.000) in both colon tissues and sera (Table 2). In addition, crocin significantly increased the concentrations of IL-4 (*p* = 0.033) and reduced those of IL-18 (*p* = 0.032) in the colon tissues (Table 2).

**TABLE 2:** The effect of crocin on levels of cytokines related to inflammation in serum and colon of mice with CRC.

		0 mg/kg	30 mg/kg
Serum	IL−1β (ng/L)	33.12 ± 0.59	12.38 ± 3.59^*^p*=0.009^
	IL−2 (ng/L)	565.27 ± 106.77	494.24 ± 81.16
	IL−4 (pg/ml)	205.31 ± 25.19	227.19 ± 23.03
	IL−6 (pg/ml)	102.02 ± 9.57	80.36 ± 8.22^*^p*=0.002^
	IL−15 (ng/L)	130.45 ± 19.95	243.98 ± 51.19^*^p*=0.000^
	IL−17 (pg/ml)	113.54 ± 10.15	88.72 ± 16.58^*^p*=0.027^
	IL−18 (pg/ml)	170.71 ± 25.90	161.79 ± 16.28
	TNF−α (ng/L)	686.61 ± 91.87	477.86 ± 56.38^*^p*=0.011^
	IFN−α (pg/ml)	91.27 ± 16.48	323.82 ± 58.17^*^p*=0.000^
Colon	IL−1β (ng/g)	26.89 ± 2.47	19.06 ± 0.97^*^p*=0.002^
	IL−2 (ng/g)	679.72 ± 100.17	657.50 ± 113.86
	IL−4 (pg/mg)	93.45 ± 29.22	144.15 ± 29.05^*^p*=0.033^
	IL−6 (pg/mg)	117.06 ± 23.44	70.19 ± 12.51^*^p*=0.001^
	IL−15 (ng/g)	88.80 ± 25.87	170.26 ± 18.32^*^p*=0.000^
	IL−17 (pg/mg)	124.39 ± 24.38	93.35 ± 16.38^*^p*=0.016^
	IL−18 (pg/mg)	161.22 ± 21.73	126.59 ± 23.73^*^p*=0.032^
	TNF−α (ng/g)	774.36 ± 134.22	577.79 ± 50.21^*^p*=0.010^
	IFN−α (pg/mg)	33.45 ± 6.41	99.16 ± 19.75^*^p*=0.000^

Data are presented as the mean ± SD (*n* = 10) and analyzed via a one−way ANOVA test followed by post−hoc Tukey's multiple comparison tests. The significant changes compared with vehicle−treated (0 mg/kg of crocin) mice marked as *^p* value.

In the colon tissues of CRC mice, crocin down-regulated the phosphorylation of IKK (α/β) (*p* = 0.000), IκBα (*p* = 0.000) and NF-κB (*p* = 0.000) and the expression of DOCK1 (*p* = 0.000), but up-regulated the expression of UBXN1 (*p* = 0.000). All these results were consistent with those detected in UC mice. Furthermore, crocin increased the expression of Bax (*p* = 0.000) and reduced the expression of Bcl-2 (*p* = 0.000) in the colon tissues of CRC mice (Figure 4C).

### Theoretical Results

**FIGURE 5:**
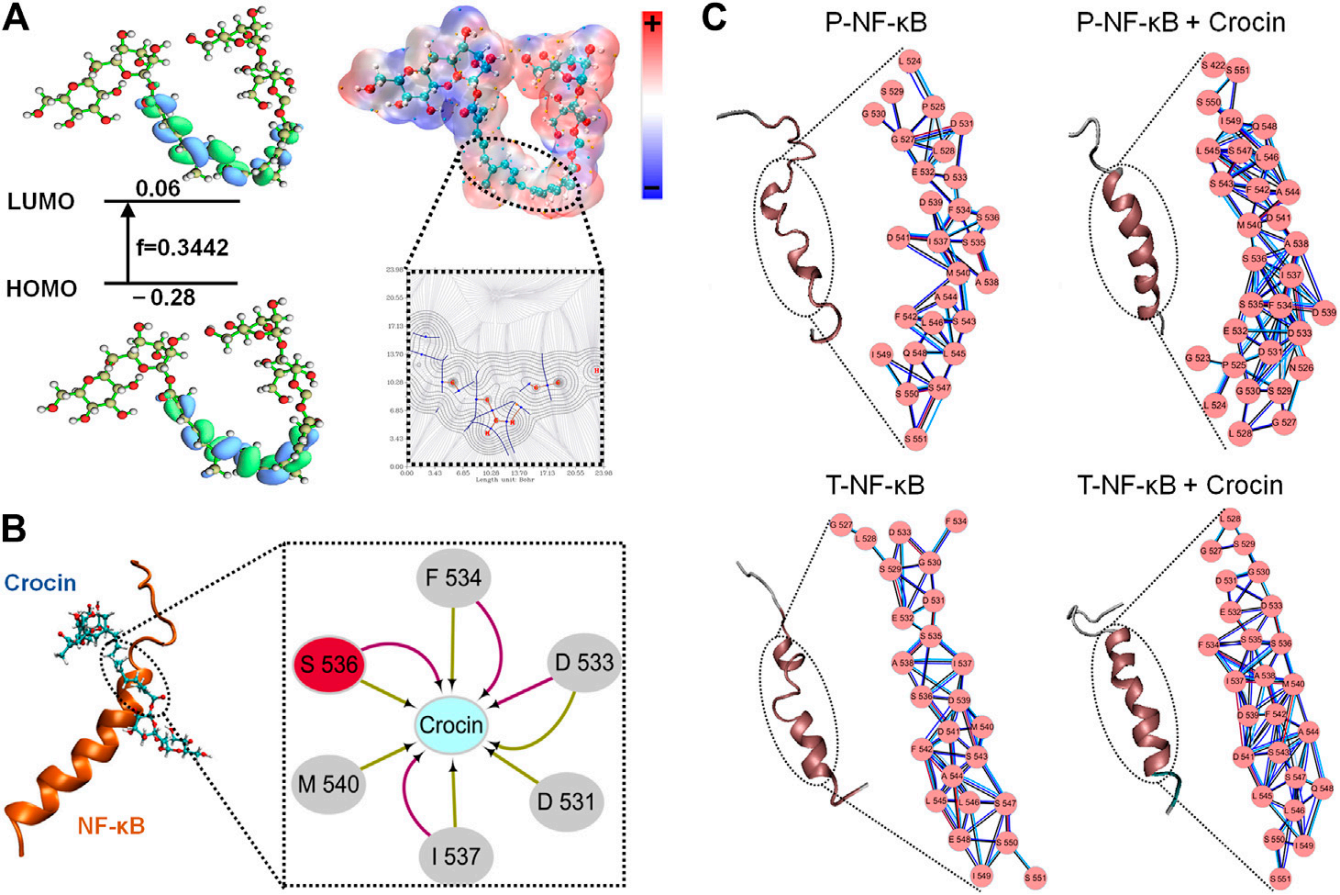
**(A)** The HOMO and LUMO orbital energy and the energy gap. **(B)** Three-dimensional structures of the complex, NF-κB is shown with cartoon and Crocin is shown as stick; Two-dimensional view of the subnetwork of substrate (Crocin) for NF-κB. **(C)** The conformational changes and detailed close-up of the network for T-NF-κB, T-NF-κB + Crocin, P-NF-κB, P-NF-κB + Crocin.

The estimated free energy of crocin binding to P-NF-κB is −7.66 kcal/mol, while the free energy of crocin binding to T-NF-κB is -6.56 kcal/mol. Seen from Figure 5A, it can be seen that LUMO orbits of crocin. LUMO component of crocin was in the center. This indicated that crocin may interact with NF-κB in the center part. Figure 5B listed the docking pose of crocin to NF-κB. F534, S536, D533, D531, I537 and M540 were important residues for crocin binding. The cluster network analysis (Figure 5C) was displayed as a detailed map of the residue interaction network. Compared with the T-NF-κB + crocin (Figure 5C), P-NF-κB + crocin could form a larger and denser cluster. MM-PBSA calculations also showed that the free energy of binding of P-NF-κB + crocin complex was lower than that of T-NF-κB +crocin complex.

## Discussion

UC is listed by the World Health Organization as one of the most refractory diseases in modern society (Chen et al., 2017a), and CRC is listed as the third most common malignant cancer in the world (Kee et al., 2019). UC may promote the development of cancer through the release of pro-inflammatory cytokines. Crocin has been confirmed to prevent or treat colitis due to its anti-inflammatory effects (Rezaei et al., 2019), and to have antitumor activity against CRC (Amerizadeh et al., 2018). However, this study is the first to confirm that the anti-UC and anti-CRC effects of crocin in mice occur via its modulation of the NF-κB-mediated inflammatory response.

In UC mice, crocin protected the colon tissue from DSS damage via its suppression of the production of various cytokines, especially ILs. Encouragingly, crocin also markedly reduced the numbers and size of colorectal tumors in CRC mice, and this was shown to occur via the anti-inflammatory activity of crocin, as evidenced by its influencing of ILs, especially those of IL-6, in the colon tissues and sera. Hyperelevated concentrations of IL-6 are consistently observed in UC and CRC patients (Waldner et al., 2012; Francescone et al., 2015). IL-6 up-regulates the expression of intercellular adhesion molecule one, a key adhesion molecule in inflammatory bowel disease (IBD), by inducing the activation of NF-κB. Thus, IL-6 contributes to the occurrence of IBD, including UC (Mudter and Neurath, 2007). Furthermore, IL-6 is considered to participate in the formation of sporadic CRC because of its trans-signaling ability (Waldner et al., 2012). In IBD, the expression levels of IL-6/soluble IL-6 receptor (sIL-6R) complexes are increased, which in turn mediate the activation of signal transducer and activator of transcription-3 (STAT3); as a result, the IL-6/STAT3 pathway can stimulate the proliferation of premalignant intestinal epithelial cells, thereby promoting tumorigenesis (Francescone et al., 2015; Luo and Zhang, 2017). UC can also cause tumorigenesis via the release of IL-6 (Lu and Zhao, 2020), IL-6 promotes the development of secondary tumors by increasing the invasiveness of colon cancer cells or promoting angiogenesis (Knupfer and Preiss, 2010). Crucially, crocin markedly decreased the concentrations of IL-6 in the sera and colon tissues of both UC mice and CRC mice.

IL-6 can also drive the secretion of IL-17 (Beriou et al., 2009). IL-17 helps to trigger monocytes or macrophages to produce the inflammatory factor TNF-α, a high-level driver of intestinal inflammation, further promoting the development of CRC (Mager et al., 2016; Asadi et al., 2017). TNF-α can stimulate the production of IL-1β, which increases the concentrations of iNOS and COX-2 (Roth-Isigkeit et al., 2001; Lee et al., 2017a). Interestingly, as a feedback loop, IL-1β can also trigger the production of IL-6 and stimulate Th17 cells (Roth-Isigkeit et al., 2001; Liao et al., 2015). IL-6 may thus play a key role in crocin-mediated anti-UC and anti-CRC effects.

Hyper-expression of NF-κB p65 has been noted in the colonic mucosa of UC patients (Yu et al., 2011). NF-κB signaling and its subsequent expression of proinflammatory cytokines are also known to play a key role in the pathogenesis of UC (Saber et al., 2019), and NF-κB activation can support tumorigenesis by promoting cell proliferation and angiogenesis, inhibiting cell apoptosis, and promoting cell invasion and metastasis (Patel et al., 2018). After IKK acts, IκBα linked to NF-κB is phosphorylated, then polyubiquitinated, and subsequently degraded by the proteasome (Park and Hong, 2016). The phosphorylated residues can get electrons more easily and thence become chemically more active (Han et al., 2017). From the network analyses, it can be concluded phosphorylated residues on S536 may enhance the protein-protein interactions which may influence the conformational changes in the secondary structure. Activated NF-κB undergoes nuclear translocation, mediates the transcription of various target genes, and directly or indirectly triggers changes in the concentrations of inflammatory factors (Park and Hong, 2016). For example, activated NF-κB triggers the activation of proinflammatory cytokines, such as TNF-α, IL-6 and IL-1β (He et al., 2019).

In UC mice, NF-κB signaling is responsible for the increased concentrations of COX-2 and iNOS (Chen et al., 2017b; Zaidi and Wine, 2018; He et al., 2019). COX-2 can participate in the inflammatory response by catalyzing the synthesis of prostaglandins (He et al., 2019), while iNOS is key to the generation of large concentrations of nitric oxide (NO), which relaxes blood vessels and increases vessel permeability, thereby increasing inflammation (Chen et al., 2017b; He et al., 2019). UBXN1 has a negative regulatory effect on NF-κB signaling, as evidenced by the fact that UBXN1 knockdown enhanced the TNF-α–triggered activation of NF-κB (Wang et al., 2015). The migration and invasion of many tumor cells is related to the upregulation of DOCK1, and DOCK1 can reverse the inhibition of IκBα phosphorylation (Li et al., 2019). It was thus particularly significant that crocin substantially decreased the concentrations of phosphorylated IKK (α/β), IκBα and NF-κB p65 in the colons of UC and CRC mice. In CRC cells, crocin caused apoptosis related to the mitochondrial dysfunction suggesting by the dissipation of MMP and the decrease in the Bcl-2: Bax ratio. Accordingly, NF-κB signaling can regulate the levels of Bcl-2 and Bax (Lu et al., 2016).

This study has some limitations. According to previous studies, crocin reduced the cell viability at different concentrations in different cells, such as 271.18 μM (48 h IC_50_) in HCT116 cells (Wang et al., 2020) and 3 mM (24 h IC_50_) in CT-26 cells (Amerizadeh et al., 2018). In SW480 cells, our data is consistent with previous research (Li et al., 2012). The different effective concentrations of crocin on different cells need further investigation, which may be related to the different possible mechanisms. Specifically, although the use of two mouse models allowed the anti-UC and anti-CRC effects of crocin to be confirmed, the effects of crocin on the development of CRC from UC were not shown. Furthermore, crocin may regulated the balance of intestinal microbiota (Xie et al., 2019), the relationship between the intestinal microbiota and inflammatory response during the crocin-mediated anti-UC and anti-CRC effects is worth for further study.

Overall, it was demonstrated that the potent anti-inflammatory properties of crocin protected the mouse colons against DSS-induced UC and suppressed tumorigenesis in Apc^MinC^/Gpt CRC mice. These effects were shown to be (at least partially) due to, crocin acting via the NF-κB signaling pathway to decrease the concentrations of IL-6. These data suggest that crocin or its structural analogs should be explored as possible therapeutic candidates for colorectal diseases, especially UC and CRC.

## Data Availability

The datasets presented in this study can be found in online repositories. The names of the repositories and accession numbers can be found in the article/Supplementary Material.

## Glossary

ANOVA: Analysis of variance
APC: Adenomatous polyposis coil
Bax: Bcl-2 associated X protein
BCA: Bicinchoninic acid
Bcl-2: B-cell lymphoma-2
BSA: Bovine serum albumin
COX-2: Cyclooxygenase-2
CRC: Colorectal cancer
CTRL: Control
DAI: Disease activity index
DMEM: Dulbecco’s modified Eagle’s medium
DOCK1: Dedicator of cytokinesis 1
DSS: Dextran sodium sulfate
ECL: Electro-chemi-luminescence
ELISA: Enzyme-linked immunosorbent assay
FBS: Fetal bovine serum
GAPDH: Glyceraldehyde-3-phosphate dehydrogenase
GO: Gene Ontology
H&E: Hematoxylin and eosin
HRP: Horseradish peroxidase
IBD: Inflammatory bowel disease
IFN-α: Interferon-α
IKK(α/β): Inhibitor of nuclear factor kappa-B kinase subunit (α/β)
IL: Interleukin
ILs: interleukins
iNOS: Inducible nitric oxidesynthase
IκBα: Inhibitor of nuclear factor kappa-B-α
KEGG: Kyoto Encyclopedia of Genes and Genomes
MMP: Mitochondrial membrane potential
MTT: 3-(4,5-dimethyl-2-thiazolyl)-2, 5-diphenyl-2-H-tetrazolium bromide
NF-κB: Nuclear factor kappa-B
NO: Nitric oxide
P-: Phospho
PBS: Phosphate buffered saline
PPAR-γ: Peroxisome proliferator-activated receptor γ
SASP: Sulfasalazine
SD: Standard deviation
sIL-6R: Soluble interleukin-6 receptor
STAT3: Signal transducer and activator of transcription-3
T-: Total-
TNF-α: Tumor necrosis factor-α
UBXN1: UBX domain-containing protein 1
UC: Ulcerative colitis
