# Supplementary material for: The Protection of Crocin Against Ulcerative Colitis and Colorectal Cancer via Suppression of NF-κB-Mediated Inflammation
Source: Front Pharmacol. 2021 Mar 18;12:639458. doi: 10.3389/fphar.2021.639458 (PMC8025585; doi:10.3389/fphar.2021.639458)
Supplement: Supplementary file 1 [file Image1.tif]

Frontiers | The protection of crocin against ulcerative colitis and colorectal cancer via suppression of NF-κB-mediated inflammation | Pharmacology


- About
- Journals
- Research Topics
- Articles
- More

Submit

My Frontiers

Office

- TSOF
  - TSOF
  - Article Production

Typesetter 3

frontiersproduction@tnq.co.in

- Profile
- Settings & Privacy
- Help Center
- Logout

Submit

**Impact Factor 4.225** | **CiteScore 5.0**More on impact ›

|  |  |
| --- | --- |
| Frontiers in Pharmacology | Inflammation Pharmacology |

Toggle navigation


Section


- (current)Section
- About
- Articles
- Research topics
- For authors 
  - Why submit?
  - Fees
  - Article types
  - Author guidelines
  - Review guidelines
  - Submission checklist
  - Contact editorial office
  - Submit your manuscript
- Editorial board

- *Article alerts*

Articles


**Suggest a Research Topic >**

- 47
  total views

 View Article Impact

**Suggest a Research Topic >**

##### SHARE ON

- Facebook

  0
- Twitter

  0
- LinkedIn

  0
- AddThis

  New


## Original Research ARTICLE

Front. Pharmacol.
| doi: 10.3389/fphar.2021.639458

# The protection of crocin against ulcerative colitis and colorectal cancer via suppression of NF-κB-mediated inflammation Provisionally accepted The final, formatted version of the article will be published soon. **Notify me**

Shanshan Teng1, 
Jie Hao1, 
Hui Bi2, 
Congcong Li1, 
Yongfeng Zhang1, 
Yaqin Zhang1, 
 Weiwei Han1\* and  Di Wang1\*

- 1School of Life Sciences, Jilin University, China
- 2Department of Anesthesiology, Hospital of Stomatology, Jilin University, China

Background: In China, the incidence of ulcerative colitis (UC) is increasing every year, but the etiology of UC remains unclear. UC is known to increase the risk of colorectal cancer (CRC). The aim of this study was to investigate the protective effects of crocin against UC and CRC in mouse models.  
Methods: Crocin was used to treat the dextran sodium sulfate (DSS)-induced UC mice for 3 weeks, and ApcMinC/Gpt mice with CRC for 8 weeks. Proteomics screening was used to detect changes in the protein profiles of colon tissues of UC mice. Enzyme-linked immunosorbent assays and western blot were used to verify these changes.  
Results: Crocin strongly reduced the disease activity index scores of UC mice, and improved the pathological symptoms of the colonic epithelium. The anti-inflammatory effects of crocin were indicated by its regulation of the activity of various cytokines, such as interleukins, via the modulation of nuclear factor kappa-B (NF-κB) signaling. Crocin significantly suppressed tumor growth in ApcMinC/Gpt mice and ameliorated pathological alterations in the colon and liver, but had no effects on spleen and kidney. Additionally, crocin significantly decreased the concentrations of interleukins and tumor necrosis factor-α in the sera and colon tissues, suggesting its anti-inflammatory effects related to NF-κB signaling. Finally, 12-h incubation of SW480 cells with crocin caused cell cycle arrest, enhanced the apoptotic rate, promoted the dissipation of mitochondrial membrane potential, and the over-accumulation of reactive oxygen species. From the theoretical analyses, phosphorylated residues on S536 may enhance the protein-protein interactions which may influence the conformational changes in the secondary structure of NF-κB.  
Conclusion: The protective effects of crocin on UC and CRC were due to its suppression of NF-κB-mediated inflammation.

Keywords: 
Crocin, ulcerative colitis, colorectal cancer, anti-inflammation, antitumor, NF-κB

Received: 09 Dec 2020;
Accepted: 11 Feb 2021.

Copyright: © 2021 Teng, Hao, Bi, Li, Zhang, Zhang, Han and Wang. This is an open-access article distributed under the terms of the Creative Commons Attribution License (CC BY). The use, distribution or reproduction in other forums is permitted, provided the original author(s) and the copyright owner(s) are credited and that the original publication in this journal is cited, in accordance with accepted academic practice. No use, distribution or reproduction is permitted which does not comply with these terms.

\* Correspondence: 
  
 Prof. Weiwei Han, Jilin University, School of Life Sciences, Changchun, 130012, Jilin Province, China, weiweihan@jlu.edu.cn   
 Prof. Di Wang, Jilin University, School of Life Sciences, Changchun, 130012, Jilin Province, China, jluwangdi@jlu.edu.cn

Write a comment...

Add

##### COMMENTARY

##### ORIGINAL ARTICLE

##### People also looked at

## Galectin-3, Possible Role in Pathogenesis of Periodontal Diseases and Potential Therapeutic Target: A Mini Review

Milica Velickovic, Aleksandar Nebojša Arsenijević, Aleksandar Acovic, Dragana Arsenijevic, Jelena Milovanovic, Jelena D Dimitrijevic, Zeljko Todorovic, Marija Milovanovic, Tatjana Kanjevac and Nebojsa Nikola Arsenijevic

## Pharmacologic Targeting of BET Proteins Attenuates Hyperuricemic Nephropathy in Rats

Chongxiang Xiong, Jin Deng, Xin Wang, Xiaofei Shao, Qin Zhou, Hequn Zou and Shougang Zhuang

## The Psychoactive Agent Crocin Can Regulate Hypothalamic-Pituitary-Adrenal Axis Activity

Sara Asalgoo, Mahdi Tat, Hedayat Sahraei and Gila Pirzad Jahromi

## Chronic Inhibition of mROS Protects Against Coronary Endothelial Dysfunction in Mice With Diabetes

Hang Xing, Zhiqi Zhang, Guangbin Shi, Yixin He, Yi Song, Yuhong Liu, Elizabeth O. Harrington, Frank W. Sellke and Jun Feng

## PDE9 inhibitor PF-04447943 attenuates DSS-induced colitis by suppressing oxidative stress, inflammation and regulating T-cell polarization

Mohammad Nasiruddin Rana, Jie Lu, Enfu Xue, Jingjing Ruan, Yuting Liu, Lejun Zhang, Rana Dhar, Yajun Li, Zhengqiang Hu, Jie zhou, Wangqian Ma and Huifang Tang

**Suggest a Research Topic >**

×

#### Supplementary Material

  

There is no supplementary material currently available for this article

Loading supplemental data...

  

|  | File Name |  |
| --- | --- | --- |
|  | Table 1.docx |  |
|  | Table 2.docx |  |
|  | Image 1.TIF |  |
|  | Image 2.TIF |  |

  

Close

- About Frontiers
- Institutional Membership
- Books
- News
- Frontiers' social media
- Contact
- Careers
- Submit
- Newsletter
- Help Center
- Terms & Conditions
- Privacy Policy

© 2007 - 2021 Frontiers Media S.A. All Rights Reserved

### Privacy Preference Center

Our website uses cookies that are necessary for its operation. Additional cookies are only used with your consent. These cookies are used to store and access information such as the characteristics of your device as well as certain personal data (IP address, navigation usage, geolocation data) and we process them to analyse the traffic on our website in order to provide you a better user experience, evaluate the efficiency of our communications and to personalise content to your interests. Some cookies are placed by third-party companies with which we work to deliver relevant ads on social media and the internet. Click on the different categories' headings to change your cookie preferences. Click on "More Information" if you wish to learn more about how data is collected and shared.
More information

### Manage Consent Preferences

#### Strictly Necessary Cookies

Always Active

These cookies are necessary for the website to function and cannot be switched off in our systems. They are usually only set in response to actions made by you which amount to a request for services, such as setting your privacy preferences, logging in or filling in forms. You can set your browser to block or alert you about these cookies, but some parts of the site will not then work. These cookies do not store any personally identifiable information.

#### Analytics Cookies

Analytics Cookies

These cookies allow us to count visits and traffic sources so we can measure and improve the performance of our site. They help us analyse which pages are the most and least popular and see how visitors move around the site.    All information these cookies collect is aggregated and therefore anonymous.

#### Functional Cookies

Functional Cookies

These cookies enable the website to provide enhanced functionality and personalisation. They may be set by us or by third party providers whose services we have added to our pages. If you do not allow these cookies then some or all of these services may not function properly.

#### Advertising Cookies

Advertising Cookies

These cookies may be set through our site by our advertising partners. They may be used by those companies to build a profile of your interests and show you relevant adverts on other sites.    They do not store directly personal information, but are based on uniquely identifying your browser and internet device. If you do not allow these cookies, you will experience less targeted advertising.

### Back Button Performance Cookies

Vendor Search  Search Icon

Filter Icon

Clear

checkbox label label

Apply Cancel

Consent Leg.Interest

checkbox label label

checkbox label label

checkbox label label

Confirm My Choices
