# Supplementary material for: The Protection of Crocin Against Ulcerative Colitis and Colorectal Cancer via Suppression of NF-κB-Mediated Inflammation
Source: Front Pharmacol. 2021 Mar 18;12:639458. doi: 10.3389/fphar.2021.639458 (PMC8025585; doi:10.3389/fphar.2021.639458)
Supplement: Supplementary file 3 [file table1.docx]

**Table S1.** The effect of crocin on differential proteins of mice with UC.

| Number | Gene names | CTRL vs. 3.0% DSS | | Crocin (30 mg/kg) vs. 3.0% DSS | Number | Gene names | CTRL vs. 3.0% DSS | Crocin (30 mg/kg) vs. 3.0% DSS |
| --- | --- | --- | --- | --- | --- | --- | --- | --- |
| 1 | Serping1 | 0.0083 | 0.0060 | | 35 | Mcm5 | 2.40 | 2.15 |
| 2 | Grem1 | 0.036 | 0.49 | | 36 | Fam175b | 2.48 | 5.07 |
| 3 | S100a9 | 0.041 | 0.27 | | 37 | Capn13 | 2.50 | 0.25 |
| 4 | Tsc22d1; Tsc22d2 | 0.067 | 0.036 | | 38 | Cyp51a1 | 2.50 | 2.96 |
| 5 | Ngp | 0.083 | 0.038 | | 39 | Engase | 2.68 | 3.19 |
| 6 | Auh | 0.10 | 0.021 | | 40 | Arfip2 | 2.70 | 2.05 |
| 7 | Itih4 | 0.12 | 0.16 | | 41 | Ncbp1 | 2.81 | 2.35 |
| 8 | Cog2 | 0.13 | 0.076 | | 42 | Rprd1b | 3.06 | 3.49 |
| 9 | Tsnax | 0.14 | 0.15 | | 43 | Lrrc40 | 3.39 | 3.84 |
| 10 | Hp | 0.15 | 0.26 | | 44 | Pabpn1; Gm20521 | 3.59 | 3.99 |
| 11 | Igkv10-96 | 0.16 | 0.065 | | 45 | Rpa3 | 3.93 | 2.84 |
| 12 | Dock1 | 0.18 | 0.11 | | 46 | Golga5 | 4.68 | 4.27 |
| 13 | Fcgr3; Fcgr2b; Fcgr2 | 0.18 | 0.39 | | 47 | Ubxn1 | 4.71 | 4.00 |
| 14 | Il1rn | 0.18 | 0.42 | | 48 | Sfxn1 | 5.44 | 6.94 |
| 15 | Serpina3n | 0.21 | 0.50 | | 49 | Vamp8 | 5.46 | 6.23 |
| 16 | Clca4a | 0.22 | 0.084 | | 50 | Nolc1 | 5.99 | 7.56 |
| 17 | Fuca1 | 0.24 | 0.40 | | 51 | Mrps15 | 6.26 | 4.97 |
| 18 | Cul2 | 0.25 | 0.14 | | 52 | Dpy30 | 6.36 | 5.17 |
| 19 | Nfs1; Gm28036 | 0.26 | 0.11 | | 53 | Srsf10; Srsf12 | 7.25 | 8.09 |
| 20 | Abi1 | 0.26 | 0.22 | | 54 | St3gal4 | 7.42 | 3.27 |
| 21 | Rbp1 | 0.27 | 0.39 | | 55 | 9530053A07Rik | 7.67 | 6.42 |
| 22 | Apoh | 0.27 | 0.024 | | 56 | Parp9 | 7.78 | 7.04 |
| 23 | Sumo1 | 0.31 | 0.20 | | 57 | Rcn2 | 8.04 | 14.32 |
| 24 | Btf3l4 | 0.42 | 0.15 | | 58 | Cbr4 | 8.31 | 7.54 |
| 25 | Chil3 | 0.42 | 0.098 | | 59 | Arap1 | 8.84 | 9.17 |
| 26 | Sec16a | 0.43 | 0.13 | | 60 | Tmed4 | 9.31 | 6.54 |
| 27 | Grn | 0.44 | 0.43 | | 61 | Pcbd1 | 11.32 | 8.29 |
| 28 | Rab27a | 0.47 | 0.13 | | 62 | Spcs1 | 13.10 | 13.99 |
| 29 | Tubb4a | 0.47 | 0.49 | | 63 | Pla2g4c | 13.20 | 3.77 |
| 30 | Sod3 | 0.49 | 2.22 | | 64 | mt-Nd2; ND2; Mtnd2 | 13.98 | 12.07 |
| 31 | Sart1 | 0.50 | 0.19 | | 65 | Acaca | 15.36 | 19.29 |
| 32 | U2surp | 2.16 | 2.26 | | 66 | Igj | 20.71 | 12.15 |
| 33 | Fam107b | 2.17 | 2.47 | | 67 | Atp5e | 21.51 | 14.34 |
| 34 | Arih1 | 2.31 | 2.58 | | 68 | Ttn | 41.37 | 35.42 |

CTRL vs. 3.0% DSS: the ratio of protein between CTRL mice and mice treated with 3.0% DSS only.

Crocin (30 mg/kg) vs. 3.0% DSS: the ratio of protein between mice treated with crocin (30 mg/kg) and mice treated with 3.0% DSS only.
